# Healthcare Settings and Infection Prevention: Today’s Procedures in Light of the “Instructions for Disinfection” Issued During the 1817 Typhus Epidemic in the Grand Duchy of Tuscany (Pre-Unification Italy)

**DOI:** 10.3390/epidemiologia6010005

**Published:** 2025-01-26

**Authors:** Davide Orsini, Maria Luisa Cristina, Anna Maria Spagnolo, Carola Minet, Marina Sartini, Roberto Parrella, Nicola Luigi Bragazzi, Mariano Martini

**Affiliations:** 1 University Museum System of Siena (SIMUS), History of Medicine, University of Siena, 53100 Siena, Italy; 2 Department of Health Sciences, University of Genoa, 16132 Genova, Italy; cristinaml@unige.it (M.L.C.); am.spagnolo@unige.it (A.M.S.); minetcarola@gmail.com (C.M.); sartini@unige.it (M.S.); mariano.martini@unige.it (M.M.); 3 Unit of Respiratory Infectious Diseases, Cotugno Hospital, Azienda Ospedaliera dei Colli, 80131 Naples, Italy; rob.parrella@gmail.com; 4 Laboratory for Industrial and Applied Mathematics (LIAM), Department of Mathematics and Statistics, York University, Toronto, ON M3J 1P3, Canada; robertobragazzi@gmail.com

**Keywords:** infections, prevention, typhus, epidemics, XIX century, Grand Duchy of Tuscany, history of medicine

## Abstract

Even today, healthcare-associated infections (HCAIs) remain the most frequent and serious complications in healthcare, with a significant clinical and economic impact. The authors of this manuscript address the causes and conditions that determine this situation and describe them in comparison with the situation in the Grand Duchy of Tuscany more than two centuries ago and with the instructions that were issued at the time to contain the typhus epidemic of 1817, increase hospital sanitation, and disinfect houses. Today, we know that a crucial element in the fight against healthcare-associated infections (HCAIs) is the definition and implementation of best care practices and other measures, according to a combined program that must be tailored to each healthcare setting. In the early nineteenth century, these approaches originated from experience and chemical knowledge that were becoming established, opening the way to the ideas and experiments of Ignác Fülöp Semmelweis and later of Joseph Lister, who traced the path for the birth of hygiene. Two centuries later the pioneering vision of the Grand Duchy of Tuscany at the beginning of the 19th century, when preventive measures in the field of public health were still backward and underdeveloped, is still enlightening and surprisingly topical.

## 1. Introduction

Between 10 and 11 April 1815, the volcano Tambora on the Indonesian island of Sumbawa, after a few days of activity, exploded with incredible violence. It was the most massive eruption of the last ten thousand years and constitutes a benchmark of the intensity of volcanic activity.

During the eruption, the volcano emitted an enormous amount of solid material and ash; some fell to the ground and into the sea, while some remained at high altitude for a long time. This material, together with the immense quantity of fumes and sulfurous gases emitted, formed a sort of atmospheric screen, which blocked part of the sun’s rays for three or four years. As a result, the Earth’s average global temperature dropped by an estimated 0.4–0.7 °C, and in some areas by even 2 or 3 °C. This phenomenon gave rise to the terms “Tambora-freeze” and “year without a summer”.

The ensuing climatic conditions affected the lives of hundreds of millions of people. Indeed, the climate changes triggered by the Tambora eruption affected the entire world at different times, but in particular the northern hemisphere, and therefore Europe, including Italy.

What does the Tambora eruption have to do with the title of this manuscript?

In addition to lowering average global temperatures, this event also had significant harmful effects on the development of edible plants, due to the increased acidity of rainwater resulting from the high concentrations of volcanic sulfur dissolved in it. Indeed, in the summer of 1816, crops ripened late, and harvests were unusually poor in both quality and quantity. Moreover, 1816 saw the outbreak of a widespread epidemic of petechial typhus, which worsened during the following year, thus compounding the problem of food shortages.

## 2. Exanthematous Typhus: A Disease Associated with Hunger, War, and Major Climate Upheavals

Exanthematous typhus, also known as petechial typhus and “febrile disease”, is an endemic infectious, contagious disease transmitted to humans by lice. Clinically, it is characterized by sudden onset, petechial rash, a typical feverish curve, and severe involvement of the nervous system. Already known in ancient times and described in the *Corpus Hippocraticum*, typhus was extensively discussed by Girolamo Fracastoro (c. 1483–1553); in his *De contagione et contagiosis morbis et eorum curatione* [1], he emphasized the peculiar aspect of the petechial rash, identifying its direct inter-human contagion and clearly distinguishing it from the plague.

Because of the frequency of its epidemics, typhus represented one of the most serious scourges of humanity, despite being much less deadly than the plague. The recurring epidemics in the Middle Ages and later centuries were often associated with a lack of hygiene, poverty, and the inability to defend against the cold. It affected and continues to specifically affect the poor, prisoners, and people forced to leave their homes because of wars and subsequent famines.

Typhus outbreaks have been problematic in every major worldwide conflict since the Peloponnesian War. Thus, understanding the drivers and preventative measures applied to historical typhus outbreaks can potentially be applied to future outbreaks, also taking into account the difficult international political situation [2].

Exanthematous typhus (also known by the names epidemic typhus, petechial typhus, dermotyphus, lice typhus, and European typhus) is a rare zoonosis caused by the bacterium Rickettsia prowazekii, transmitted to humans by the bite of certain species of lice and fleas. Unlike other diseases transmitted by fleas and lice where humans are occasional hosts, in this case, they are the main reservoir. The people most susceptible to infection are those who live in poor hygienic conditions conducive to the spread of lice, such as the homeless or in those displaced by natural disasters or even soldiers [3].

The body louse lays its eggs in the seams of clothing. The disease is spread by inoculating infected lice feces into the skin. Disinfection of clothing could have been beneficial to combating an outbreak by destroying the eggs on clothing and killing the viable bacteria in the fecally contaminated clothing, bedding, etc.

Unfortunately, the discovery of the etiologic agent of typhus dates back only to the early 20th century when Charles Nicolle (1866–1936), a bacteriologist and hygienist who was director of the Pasteur Institute in Tunis from 1904, understood the mode of transmission of exanthematous typhus, recognizing the louse as the infectious vehicle of the disease. For this discovery, he was awarded the Nobel Prize in Medicine in 1928.

Following this discovery, the search for various ways to scientifically “sterilize” people, clothing, and other possessions from lice and louse feces became a significant endeavor during WWI. In the early 20th century, even with the knowledge of louse-borne transmission of typhus, spreading disinfectants, such as carbolic acid, cresol, and lime, into rooms, train cars, etc., was considered a part of typhus control, because it would kill stray lice and kill the bacteria in louse feces; the dried feces are low-density and easily dispersed [4,5].

Typhus epidemics are often associated with malnutrition and poor harvests.

Persons who survive infection with *Rickettsia prowazekii* are immune to re-infection. However, *Rickettsia prowasekii* can lay dormant for years in the adipose tissue of recovered patients. Physiologic stress, such as malnutrition, may cause this latent infection to reactivate, a condition known as Brill–Zinsser disease, and these patients become potentially infectious once again [6,7].

## 3. Abandonment of the “Miasma Theory”, the Discovery of Chlorine, and the Chemical Revolution of Disinfection

The typhus epidemic of 1816–1817 occurred in various countries in Europe and particularly in Italy, especially in Veneto, Friuli, and the kingdom of Lombardy-Venetia, where a high mortality rate was recorded.

The typhus epidemic caused 848 deaths in Milan hospitals alone, and the number of deaths throughout Lombardy totaled 70,644 during 1817 [8].

But even the southern regions were not spared, if we consider the figure for Foggia, where the death rate rose from 50.4‰ in 1815, to 70.5‰ in 1816, settling in 1817 at 109.5‰ [9].

In the Italian peninsula, due to typhus and food shortages, the mortality level during 1811–1820 averaged 36‰ [10].

Such considerable increases in mortality rates never originate from a single factor: it is necessary for the joint action of several factors to occur at a given time. Such was the case in the period under consideration when political changes were compounded by heavy social degradation and the decay of agricultural production due to intense volcanic activity culminating in the memorable eruption of the Tambora volcano.

It was precisely during the typhus epidemic of 1816–1817 that the miasma theory was definitively superseded. In fact, since ancient times, this theory—according to which the diffusion of poisonous particles in the air came into contact with man—had been invoked to explain the spread of infectious diseases.

Formerly, aromatic substances had been burnt in public squares to ‘purify’ the air and disperse the miasmas. Similarly, the homes of the sick were washed with vinegar and the walls were whitewashed with lime, while the linen belonging to the sick was boiled with lye and rock alum and then left for days under running water [11].

Against this theory, a completely new approach emerged during the second half of the Enlightenment period as a result of discoveries in the field of chemistry; in order to prevent epidemics, a policy of sanitization and disinfection of places and people was implemented.

At the same time, the idea of a correlation between disease and the environment, work, and pollution was also gaining ground.

**Figure 1:**
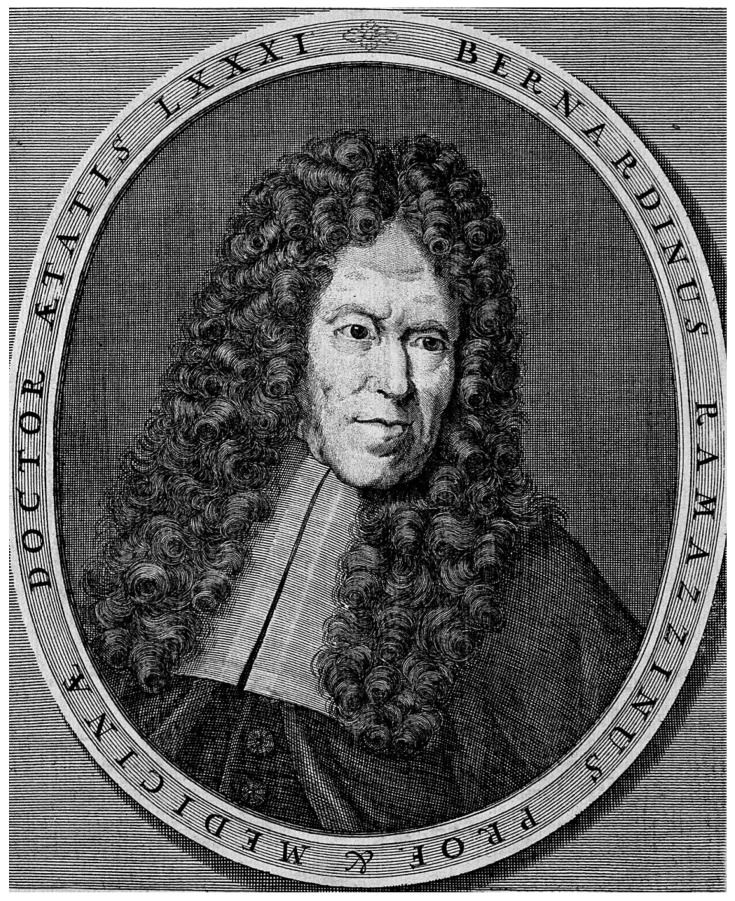
Bernardino Ramazzini (1633–1714) (Wikipedia Commons—Public Domain).

A century earlier, Bernardino Ramazzini (1633–1714) [Figure 1], who is considered the founder of occupational medicine and author of the famous *De morbis artificum diatribe* (Diseases of Workers), printed in 1745, had already written that “advances in medicine should not be made only in the clinical and physiological fields, but also in the field of public health, by observing the possible relationships between environmental factors and diseases” [12].

This idea was taken up and developed by Giacomo Barzellotti (1768–1839), a student of the anatomist Paolo Mascagni (1755–1815) at the University of Siena and the founder of the School of Forensic Medicin [13,14], which counted among its major exponents Carlo Livi (1823–1877), the great criminal anthropologist Salvatore Ottolenghi (1861–1934), Cesare Biondi (1867–1936), and Giuseppe Bianchini (1888–1973) [15].

Barzellotti wrote a veritable manifesto for the prevention of poverty [16], which he considered, together with harsh working conditions, one of the causes of the onset of diseases.

Thus began a process whereby the academic training of doctors involved exercises, internships, and experiments, starting from the dissection table, in order to acquire the necessary anatomical knowledge. Among the scientists and teachers who implemented this approach, we may mention Paolo Mascagni, who combined a wealth of knowledge with vast experience, including naturalistic and geo-mineralogical observations, work at the dissection table, and university teaching [17].

In the same period, interesting developments were taking place in botany and biology, as well as chemistry.

These prompted doctors to study the etiology of diseases, particularly epidemic diseases, which led to great advances in the second half of the 19th century, thanks to the discoveries made by Louis Pasteur (1822–1895) and Robert Koch (1843–1910).

But already by the end of the 18th century the watchword was ‘sanitize’. Chemistry, developed by Antoine-Laurent de Lavoisier (1743–1794) and his colleagues, facilitated the introduction of a new way to disinfect the air and destroy contagious substances.

In 1774, the Swedish chemist Carl Wilhelm Scheele (1742–1786) made a fundamental discovery; through the reaction of hydrochloric acid and manganese dioxide, he obtained oxidized muriatic acid, an effective disinfectant; later, he also succeeded in isolating chlorine.

**Figure 2:**
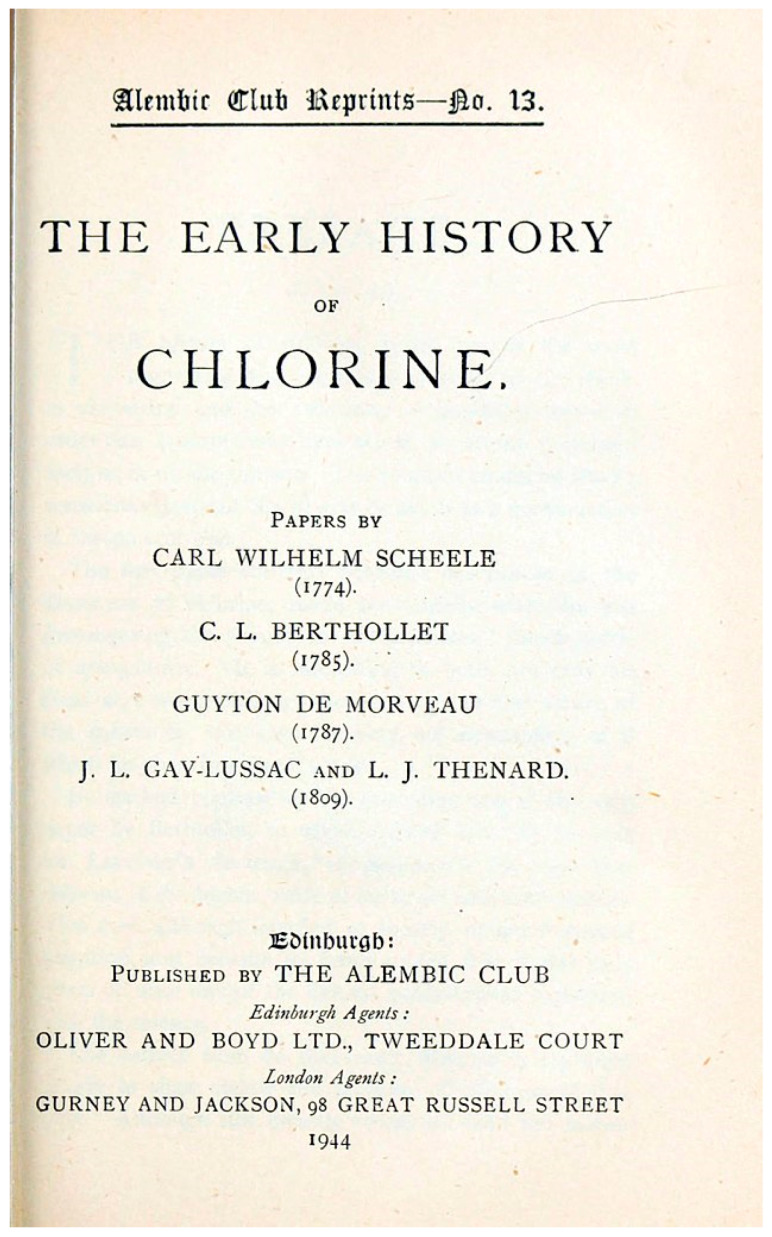
Early history of chlorine, 1944 (Wikipedia Commons—Public Domain).

In 1777, the French chemist Claude Louis Berthollet (1748–1822) bubbled chlorine with sodium carbonate (Na_2_CO_3_), obtaining a liquid with a strong bleaching power and antiseptic properties; this liquid, today known as sodium hypochlorite, he called “Javel water” [Figure 2]. 

Pierre François Percy (1754–1825), a French physician and surgeon who served as chief surgeon in Napoleon Bonaparte’s Grande Armée, used Javel water to disinfect soldiers’ wounds, achieving extraordinary results.

Again, starting from chlorine, in 1820, the French pharmacist Antoine Germain Labarraque (1877–1850) combined sodium and calcium hypochlorites to make “*liqueur de Labarraque*”, which had an even greater germicidal power than Javel water.

Infection rates were higher in hospitals and in many community environments than in other settings, owing to the high concentration of people in confined spaces and the greater possibility of transmitting pathogens from one person to another. Sodium hypochlorite and calcium hypochlorite were increasingly used to sanitize such environments; the experience of Ignác Fülöp Semmelweis [18] (1818–1865) is an example. In 1847, the Hungarian doctor, who worked in the obstetric department of a Vienna hospital, was struck by the high number of deaths due to postpartum infections. He therefore asked his students and colleagues to wash their hands with calcium hypochlorite before entering the department. Within a few months, the number of deaths due to puerperal fever decreased significantly. This proved that Semmelweis had been right, even though pathogenic germs were unknown at the time (being discovered by Louis Pasteur only in the second half of the 19th century).

A further example concerns Joseph Lister (1827–1912). Like Semmelweis, he was struck by the high incidence of hospital cases of gas gangrene, and realized that these infections, which were often lethal, were caused by the inadequate hygiene conditions in which surgical operations were performed. Having heard of Pasteur’s germ theory, Lister deduced that it was essential to keep wounds completely free of germs after operations.

For this reason, in 1865, he applied phenol to a boy’s compound fracture, which would inevitably have resulted in death from gangrene.

**Figure 3:**
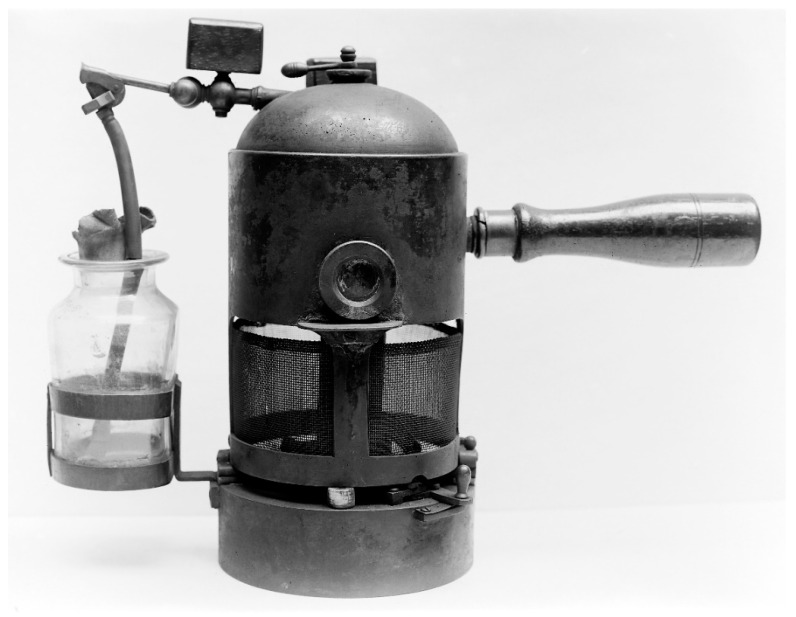
Phenol nebulizer introduced into operating theaters by Joseph Lister (image publicly available).

Thanks to the use of phenol, which was also sprayed over the entire operating field [Figure 3], the wound did not suppurate and the only side effect was burning of the skin due to the acid. Despite opposition from his colleagues, Lister’s sensational discovery ushered in the concept of antisepsis, thereby revolutionizing surgical practice [19,20].

## 4. The “Febrile Disease” That Appeared in 1817 and Disinfection by Means of the Guyton-Morveau Method

Returning to the typhus epidemic that spread to various parts of Italy in 1817, various chronicles provide information on the spread of the disease and its evolution. A booklet written by Vincenzo Chiarugi (1759–1820) provides an interesting overview of what happened in the Grand Duchy of Tuscany, and specifically mentions the *Instructions for the disinfection and isolation of hospitals* and those for *the disinfection of houses and furnishings of patients with the reigning fever* [21].

Having graduated in philosophy and medicine in 1779 at the University of Pisa, Chiarugi devoted himself to the care of the mentally ill and, together with Philippe Pinel (1745–1826), rationally addressed the problem of assistance in mental hospitals. He organized and directed the Florentine mental hospital known as Bonifazio, setting out his innovative criteria in the *Regulations of the Royal Hospitals of S. Maria Nuova and Bonifazio* (1789).

When the typhus epidemic broke out in 1817, he was appointed Superintendent of the Infirmaries of Santa Maria Nuova, his task being to organize systems of prevention and treatment of the disease based on the etiological study and course of the disease.

**Figure 4:**
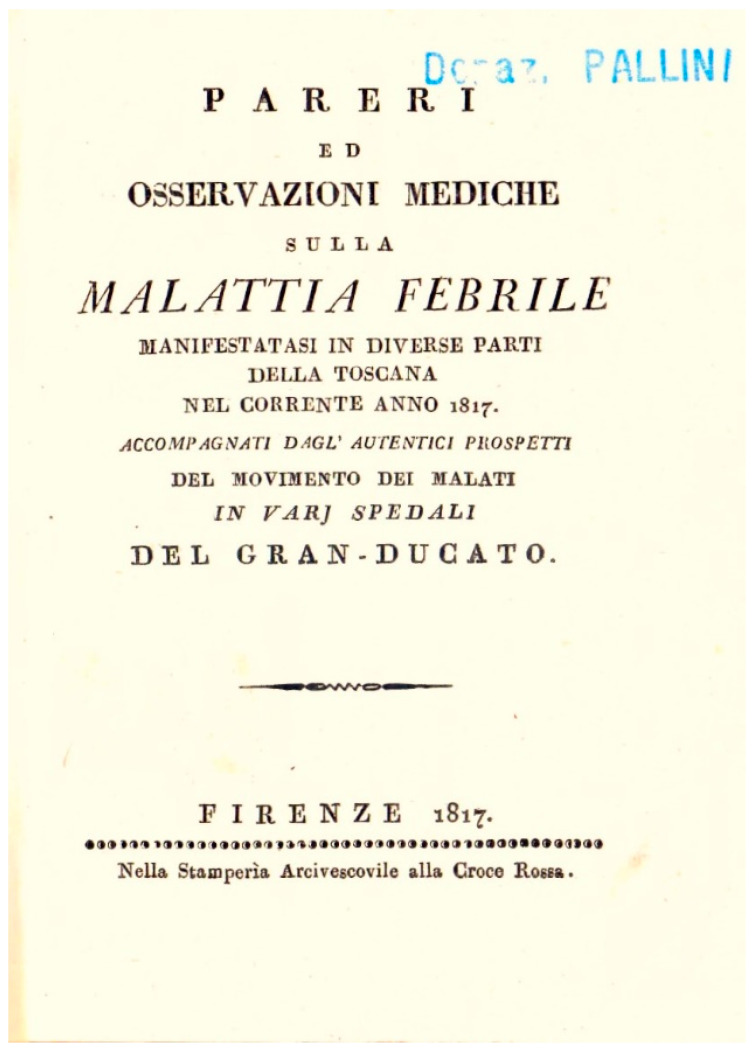
Vincenzo Chiarugi, *Opinions and medical observations on the feverish disease that manifested itself in various parts of Tuscany in the current year* 1817 (Museum of Medical Instruments—Siena University Museum System).

Published immediately after the end of the epidemic crisis, his booklet *Opinions and medical observations on the feverish disease that manifested itself in various parts of Tuscany in the current year 1817* [Figure 4] provides important evidence of the measures taken to fight the disease.

According to Chiarugi, petechial typhus was contracted “through close contact of a sick body with a healthy body” [21], thus definitively overcoming the concept of “infected air”.

Having first described the “prolegomena or precursor symptoms: headache, pain in the muscles and almost in the bones, especially in the loins and lower extremities; loss of appetite, restless sleep, total decline of physical and intellectual strength” and the “concomitant symptoms” [21], he went on to discuss treatment.

This did not differ greatly from the traditional approach: “evacuate the miasma from the body through the stomach and the skin […]; moderate the morbid reaction […]; promote evacuation of the matter reproduced in the body” [21].

What was particularly innovative, however, was the attention he devoted to the methods of disinfection of hospitals. Indeed, Chiarugi placed great trust in hospitals, which were undergoing major modernization and which he regarded not only as places of care but also as institutions where research and experimentation were to be carried out.

He wrote: “Every sick person who comes to this Hospital (Santa Lucia in Florence, intended for individuals suffering from fevers) is to deposit his or her clothes, which are to be placed in the Lazzaretto to be disinfected. […] The bed in which a sick person has lain must be purged, whatever the outcome of the disease, the straw being burnt and the linen and wool washed. The wards, or Halls, where the sick reside are to be continually ventilated and disinfected three times a day according to the Guyton-Morveau method” [21]

**Figure 5:**
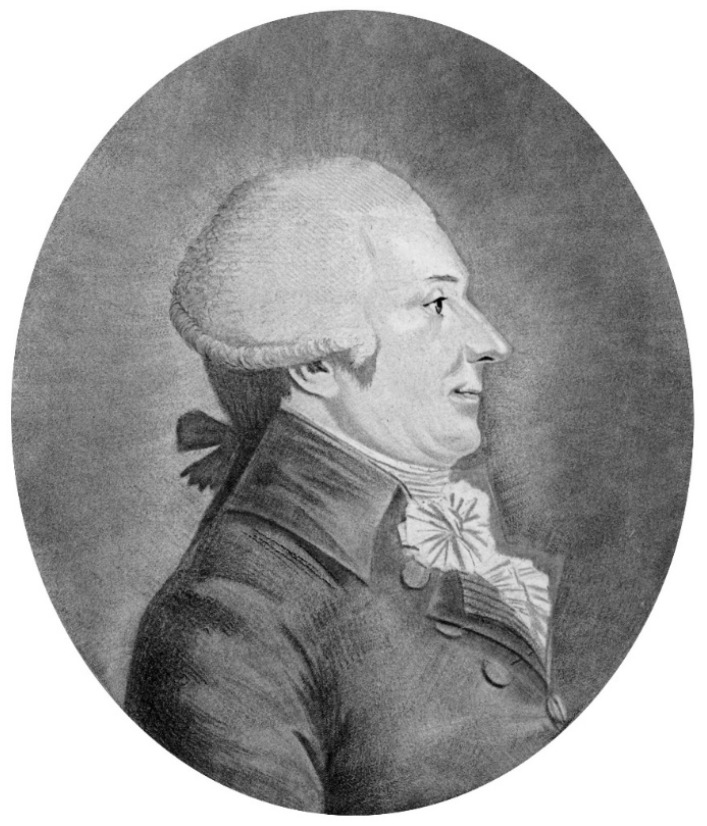
Louis Bernard Guyton de Morveau (image publicly available).

At the beginning of the 19th century, the French chemist Louis Bernard Guyton de Morveau (1737–1816) [Figure 5] described a new way of combating miasmas dissolved in the air by using chlorine vapors [22].

Morveau’s disinfection apparatus consisted of a thick-walled glass container set into a wooden frame. A large screw fixed a wooden stopper to the container. Apparatuses of this kind were normally used to demonstrate phenomena regarding the compression of air.

**Figure 6:**
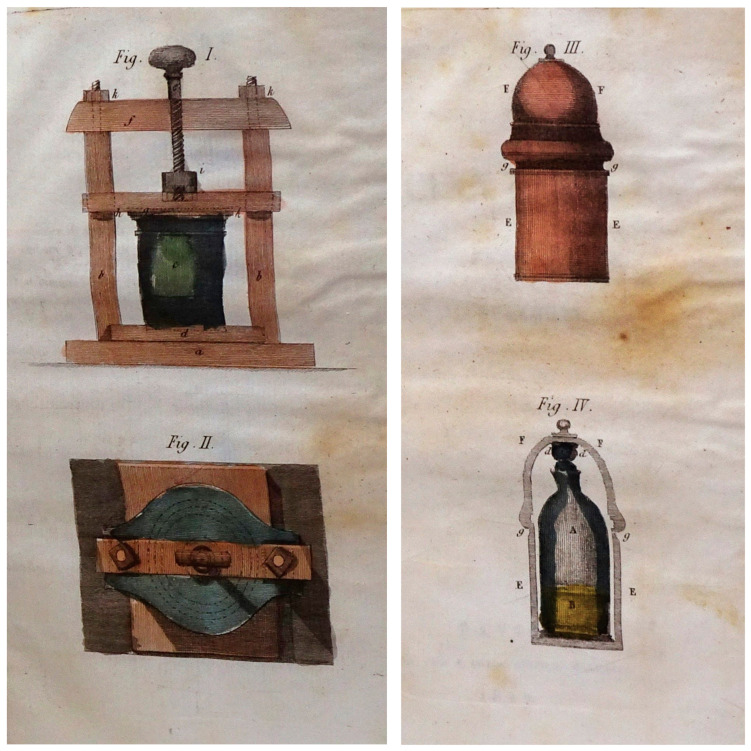
Two different models of Morveau’s disinfection device (from Guyton de Morveau, *Method of Purging Infected Airs and Protecting against All Contagious Diseases.* G. Silvestri, Milan, 1817).

Morveau’s apparatus, however, was used to prepare chlorine by reacting manganese oxide with common salt in the presence of sulfuric acid, and keeping it under moderate pressure. On opening the cap of the container slightly, the chlorine spread into the environment, acting as a disinfectant [Figure 6].

This system of disinfection, which Guyton de Morveau introduced at the beginning of the 19th century, was widely used in hospital wards between about 1805 and 1820 [23,24].

**Figure 7:**
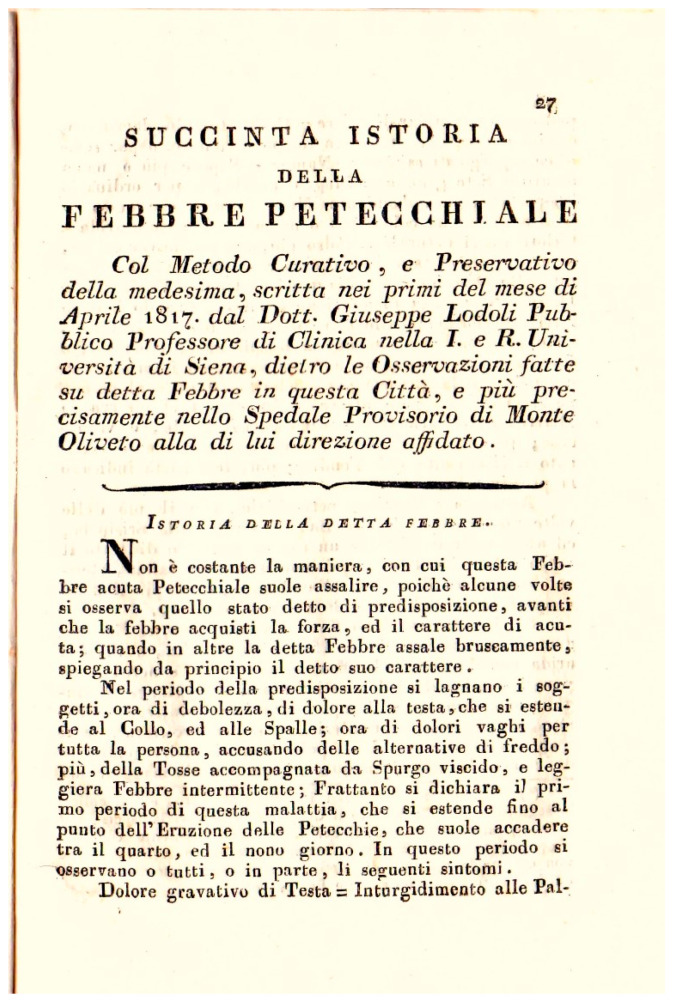
*Brief History of Petechial Fever in Siena* (Museum of Medical Instruments-Siena University Museum System).

Pages 27–31 of the above-mentioned booklet by Chiarugi also report a brief history of Petechial Fever together with a method of treating and preventing it, written at the beginning of April 1817 by Dr. Giuseppe Lodoli, Public Professor of Clinical Medicine at the I. and R. University of Siena [Figure 7].

In the booklet, Lodoli (1760–1823), who was the first director of the Sienese asylum of San Niccolò, recommends that “M. Guyton-Morveau’s disinfectant machine should be activated in the infirmaries several times a day”.

This booklet illustrates the great effort made to combat the spread of the disease in the Grand Duchy of Tuscany.

The Grand Duchy of Tuscany was an ancient Italian state that existed between 1569 and 1859. It was established by a papal bull issued by Pope Pius V on 27 August 1569, after the conquest of the Republic of Siena by the Medici dynasty, rulers of the Republic of Florence, in the final phase of the Italian wars of the 16th century.

In the manuscript “Comparative overview of the movement of the sick in the temporary hospitals of Florence, Siena and Grosseto”, which was attached to the booklet, it emerges that, thanks to the measures of prevention and disinfection, “no military garrison, none of the five convict baths, no prison, none of the detention centres or the refuges of the poor, none of the many colleges and houses of education for either sex and no convent has been attacked by this disease” [21].

## 5. Instructions for the Disinfection and Isolation of Hospitals in the First Half of the Nineteenth Century

In his booklet, Vincenzo Chiarugi also recommends maximum isolation of the sick, in order to avoid the spread of epidemics.

Among the most interesting instructions regarding hygiene practice in the second half of the 19th century is one prescribing that “any individual assigned to the immediate service of the hospital’s patients and who comes into contact with these patients is to wear an overall; this is to be worn only in the hospital itself and the individual must not leave the confines of the hospital while wearing this garment” [21]. In addition, to avoid infection through contact with objects, it is specified that “no hand-cart, stretcher or other means that has been used to transport the sick is to be used to transport out of the hospital convalescent patients or those who have recovered” [21]. The same rule was to be applied to clothing, bedding and utensils.

Regarding the disinfection of wards, he wrote that “a specific individual will be assigned to carry out muriatic fumigations three times a day in the hospital infirmaries; he will also be responsible for carrying out the same in the room designated for the disinfection of service clothing […] of doctors, surgeons, apothecaries and chaplains” [21]. Furthermore, “aromatic vinegar is to be continually evaporated in the centre of the wards; and when staff have finished their service and left the stretchers to be disinfected, they must wash their hands with vinegar diluted half and half with water” [21].

These instructions also testify to the first attempts to provide a service uniform for hospital staff, a forerunner of the modern white coat.

Very similar instructions were also given for the disinfection of houses. Specifically, Chiarugi prescribed that “once the bed, bed linen, clothes and other things used by the sick person have been washed, the rooms that have been occupied by the person are to be exposed to the action of disinfectant steam, that is, oxygenated muriatic acid gas. The person in charge of disinfection, after having placed in the room the material necessary to produce the said steam, is to close the room as tightly as possible and keep it closed for at least 12 h, or even longer in proportion to its size” [21].

On 28 June 1817, the General Office of the Communities of the Grand Duchy of Tuscany issued a note addressed to the “Gonfalonieri” (i.e., the leading citizens of the communities), in which we read the following:

“The instructions contained in the pamphlet entitled ‘Medical opinions and observations on the feverish disease that manifested itself in various parts of Tuscany in the current year 1817’, concerning the disinfection and isolation of hospitals, as well as the disinfection of the houses and furnishings of those suffering from this fever, have been recognized as beneficial to public health and have prevented the spread of the disease” [25].

## 6. Conclusions

Even today, healthcare-associated infections (HCAIs) remain the most frequent and serious complications in healthcare, with a significant clinical and economic impact.

According to the first global report by the World Health Organization (WHO) [26], the impact of HCAI implies prolonged hospital stay, long-term disability, increased resistance of microorganisms to antimicrobials, a massive additional financial burden for health systems, high costs for patients and their families, and excess deaths. In Europe, HCAIs cause 16 million extra days of hospital stay, lead to 37,000 attributable deaths, and contribute to an additional 110,000 every year. Annual financial losses are estimated at approximately EUR 7 billion, including direct costs only.

The emergence of antibiotic-resistant bacterial strains, primarily due to incorrect or excessive use of these medications, further complicates and exacerbates the impact of healthcare-associated infections (HCAIs) on public health.

Among the contributing factors to the development of healthcare-associated infections are the progressive introduction of new healthcare technologies, the prolonged use of invasive medical devices, and complex surgical procedures. Other factors relate to patient characteristics, such as increasing age [27], weakened immune systems (immunosuppression), and the presence of severe comorbidities. In this context, it is essential to control the spread of microorganisms within the hospital environment and their transmission from one patient to another. Currently, the standard method for disinfection in healthcare settings is chemical disinfection., particularly using products based on chlorine, oxygen, [28], alcohol, etc. Other, more innovative methods that complement the standard cleaning and disinfection procedures involve the use of ultraviolet (UV) rays which provide effective results in both the reduction in hygiene failures and in controlling environmental contamination by high-concern microorganisms [29]. However, disinfectants are not always used strictly according to the label, making them less effective in disinfection. Among the pathogens involved in HAIs, it has been observed that bacteria can adhere to surfaces and aggregate, forming a complex structure, called biofilm, which makes them much less sensitive to external agents.

Innovative strategies for the treatment of biofilm employ multitarget nano-platforms, structures engineered at the nanometer scale used to carry and deliver active agents, capable of degrading bacterial biofilm and killing dispersed cells to reduce diverse types of infection, allowing safer treatment paths.

The prevention and control of healthcare-associated infections (HCAIs) in all healthcare settings are essential measures to reduce the impact of these infections and, more broadly, to curb the spread of antibiotic-resistant microorganisms [30,31,32].

Today, we know that a crucial element in combating healthcare-associated infections (HCAIs) is the definition and implementation of best care practices and other measures, according to an integrated program that must be tailored to each healthcare setting.

Therefore, in addition to the already mentioned hand hygiene (which remains one of the most important and effective measures), efforts should also focus on reducing unnecessary diagnostic and therapeutic procedures, the correct use of antibiotics and disinfectants, and implementing measures related to maintaining asepsis during invasive procedures, the appropriate use of antibiotic prophylaxis, and the administration of recommended vaccinations [26,33,34,35].

Another important measure is the surveillance of infections through the ongoing implementation of national and international protocols, such as National Healthcare Network Safety Surveillance, Surveillance of healthcare-associated infections and prevention indicators in European intensive care units, Sorveglianza delle infezioni del sito chirurgico (SNICh), Sorveglianza attiva Prospettica delle Infezioni Nosocomiali nelle Unità di Terapia Intensiva (SPIN-UTI), national and local prevalence studies, and the monitoring of healthcare-associated infections (HAIs). These activities provide homogeneous, representative, timely, and adequate data on the spread of infections, which are essential for their control and management [36,37,38,39,40,41]. Despite the historical and pioneering understanding of the importance of measures to contain the spread of microorganisms, the inadequate application of environmental hygiene practices and infection prevention and control measures in healthcare settings (such as isolation protocols, hand hygiene compliance, etc.) remains a critical issue, and a lot of work still needs to be done in this regard in various healthcare settings, including staff training.

It is extremely interesting that measures of prevention and disinfection were considered fundamental to avoiding the spread of typhus many decades before the discoveries made by Louis Pasteur and Robert Koch led to a broader understanding of infections, and exactly 30 years before Semmelweis’s decision to oblige staff entering the obstetrics department of the Vienna hospital to wash their hands with a calcium hypochlorite solution (a measure that was actually contested by his medical colleagues).

Today, two centuries later, the pioneering vision of the Grand Duchy of Tuscany at the beginning of the 19th century, a time when preventive measures in the field of public health were still backward and underdeveloped, is still enlightening and surprisingly topical.

## Data Availability

No new data were created or analyzed in this study. Data sharing is not applicable to this article.
